# Understanding the uneven spread of COVID-19 in the context of the global interconnected economy

**DOI:** 10.1038/s41598-021-04717-3

**Published:** 2022-01-13

**Authors:** Dimitrios Tsiotas, Vassilis Tselios

**Affiliations:** 1 Department of Regional and Economic Development, Agricultural University of Athens, Nea Poli, 33100 Amfissa, Greece; 2 Department of Economic and Regional Development, Panteion University of Social and Political Sciences, 17671 Athens, Greece

**Keywords:** Environmental social sciences, Diseases

## Abstract

The worldwide spread of the COVID-19 pandemic is a complex and multivariate process differentiated across countries, and geographical distance is acceptable as a critical determinant of the uneven spreading. Although social connectivity is a defining condition for virus transmission, the network paradigm in the study of the COVID-19 spatio-temporal spread has not been used accordingly. Toward contributing to this demand, this paper uses network analysis to develop a multidimensional methodological framework for understanding the uneven (cross-country) spread of COVID-19 in the context of the globally interconnected economy. The globally interconnected system of tourism mobility is modeled as a complex network and studied within the context of a three-dimensional (3D) conceptual model composed of network connectivity, economic openness, and spatial impedance variables. The analysis reveals two main stages in the temporal spread of COVID-19, defined by the cutting-point of the 44th day from Wuhan. The first describes the outbreak in Asia and North America, the second stage in Europe, South America, and Africa, while the outbreak in Oceania intermediates. The analysis also illustrates that the average node degree exponentially decays as a function of COVID-19 emergence time. This finding implies that the highly connected nodes, in the Global Tourism Network (GTN), are disproportionally earlier infected by the pandemic than the other nodes. Moreover, countries with the same network centrality as China are early infected on average by COVID-19. The paper also finds that network interconnectedness, economic openness, and transport integration are critical determinants in the early global spread of the pandemic, and it reveals that the spatio-temporal patterns of the worldwide spreading of COVID-19 are more a matter of network interconnectivity than of spatial proximity.

## Introduction

The COVID-19 coronavirus disease (SARS-CoV-2) is the new pandemic that emerged in December 2019, in Wuhan city, China, and ever since has been rapidly spread around the world, causing cascading deaths to humanity, vast pressures on the national public health systems, and uncertainty about the future of the global and national economies^1,2^. The peculiar biological, epidemiologic, and spreading COVID-19 features^3–7^ have equipped this disease with the threat properties of a pandemic, resulting to the global awareness and outbreak of research. It has been almost two years since its emergence in Wuhan, and the COVID-19 pandemic has already become a prime concern and priority for the scientific community^2,5^. According to the Google Scholar academic database^8^, the search of the keyword “COVID-19” yields approximately 4.35 m (million) results, whereas other keywords referring to established research fields (many of which enjoy centuries of scientific research) yield a comparable number of results, such as the cases of “gravity” (4.06 m results), “cancer” (6.05 m results), “networks” (5.64 m results), “economy” (4.83 m results), “electric” (6.69 m results), “health” (6.93 m results), “space” (7.3 m results), and “science” (8.44 m results). In the already vast COVID-19 literature, someone can observe three major research strands (directions) in the study of the pandemic’s spread. The first concerns the patterns and causes^9–13^, the second strand regards the effects^2,14–16^, and the third one the management, treatment, and cure^17–20^ of the COVID-19 spread. These directions involve applications in various fields, such as individual and public health, medicine and clinical research, pharmacy, policy, economy, society, education, communication, transportation, environment, geography, and many others. According to all these diverse approaches, relevant scientific research is multidisciplinary, broad, and complex^2,5,7,21–23^, illustrating the linkage of COVID-19 with all aspects of everyday life, along with the intention of the humanity to get ahead in the fight against the pandemic.

Although a thorough review of the vast and multidisciplinary COVID-19 literature is an ongoing and future challenge for epistemology researchers, the relationship between the pandemic’s spreading and the interconnected socioeconomic structure of the modern world is more than evident in the literature^2,12,16,24–27^. At the microscopic level, the relationship between individual connectivity and COVID-19 spread (transmission) builds on clinical and epidemiologic terms. This approach has already enjoyed fruitful research contributing to the understanding and pandemic management^28–30^. In macroscopic terms, the effect of interconnectedness on the pandemic's spread is mainly studied on a dual basis, either within or between countries (in a cross-country framework). The first approach covers all topics of interest about the pandemic, such as regional outbreaks and spread due to imported cases^31,32^, mobility, and travel restrictions^14,15^, the effects of lockdown^17,33^, and others^4,34^. Even when is implemented on an international scale, the within-countries approach conceives interconnectedness as an intrinsic property of countries, interpreting the uneven spread of the pandemic based on comparing such intrinsic properties between countries. In addition, the second (cross-country) approach conceptualizes the spread-channels (links) of the pandemic between countries by configuring variables or indicators approximating aspects of interconnectedness, such as the number of tourists, geographical distance, exports per capita, motorway density, etc.^35–37^.

However, interconnectedness becomes better conceivable in the context of communication theory^38,39^, according to which the infection of COVID-19 is the result of virus transmission between two individuals^3,40^, namely an elementary communication structure called either link or edge^41^. Building on such pair-wise configuration, the complex COVID-19 virus transmission system can excellently represent a network structure and be modeled as a graph, as conceived by network science^41–44^, at any spatial and functional level. In the literature, this cognition appears to become deeper. For instance, the authors of^45^ applied social network analysis to track the spread of COVID-19 in India and found that international travels were determinative of the pandemic outbreak in the country. On the same basis, the work of^46^ studied a social network of the COVID‑19 spread in South Korea, finding that the topology of the infection network was dependent on the policy measures applied by the government to control the pandemic. The paper of^47^ constructed a multilayer network of social connectivity to study the COVID-19 epidemic spread in Brazil and the country’s potential to manage the effect of the pandemic, proposing the increase of social isolation (up to a lockdown) as the best option to preserve the functionality of the healthcare system capacity. The work of^48^ developed a multilayer network methodology of the relationship between socio-cultural and economic characteristics to identify the pandemic spreaders, resulting in a classification of the countries according to their socioeconomic, population, Gross Domestic Product (GDP), health, and air connectivity attributes. The authors of^49^ developed a network model of social contacts between individuals, calibrating their model on data about COVID-19 spread in Wuhan (China), Toronto (Canada), and the Italian Republic, using a Markov Chain Monte Carlo (MCMC) optimization algorithm. The results showed a good fitting of the network model to empirical data and provided a tool for the health authorities to plan control measures against the pandemic. The paper of^13^ used social network analysis to identify the clusters of the COVID-19 global spread and observed that the pandemic spread followed a route from China to West Asia, Europe, North America, and South America according to three classifications. The authors of^50^ developed a SAIR (Susceptible-Asymptomatic-Infected-Removed) model on social networks to study the spread of COVID-19 and examined the effectiveness of policy measures aiming to control the pandemic. Also, the authors of^51^ used agent-based simulations based on the SEIR (susceptible–exposed–infectious–recovered) model to show that two network intervention strategies dividing or balancing social groups can substantially reduce transmission while sustaining economic activities. On a similar framework, the authors of^52^ developed a contact network to study the city-level transmission using an infectious agent spread (SEIR) model, showing that precise knowledge of epidemic transmission parameters is not required to build an informative model of the spread of disease.

Further, the work of^53^ studied the impact of human mobility networks on the COVID-19 onset in 203 different countries. The authors used exponential random graph models to perform an analysis of the country-to-country global spread of COVID-19. The analysis showed that migration and tourism inflows were factors increasing the probability of COVID-19 case importations. The authors of^54^ studied a knowledge network model of semi-supervised statistical learning constructed on aerial mobility data of Hong Kong and Wuhan. The purpose of the study was to determine the early identification of infectious disease spread via air travel and align the need to keep the economy working with open connections and the different dynamics of national pandemic curves. The work of^55^ applied a network inference approach with sliding time windows to capture the spatiotemporal influence of infections and trace the spread of the pandemic in New York and the USA. The paper of^56^ employed an agent-based model to nearly 1.6 million firms in Japan and simulated the pandemic’s propagation, where they evaluated lockdown scenarios of Tokyo, Japan. On a similar conceptualization, the authors of^57^ examined how the economic effects of lockdowns in different regions interact through the supply network of 1.6 million firms in Japan. The analysis showed that a region’s upstream-ness, the intensity of loops, and supplier substitutability in supply chains with other regions largely determine the economic effect of the region's lockdown. The paper of^58^ investigated the impact of COVID-19 on global air transportation at different geographical scales, namely worldwide, international country, and domestic airport networks, for representative cases, and found that the impacts of the pandemic were concordant, in intensity, with the geographical scale. The work of^59^ developed a complex network of COVID-19 correlations between 122 countries and empirically investigated the network connectedness influencing macroeconomic and social factors. The analysis showed that population density, economic size, trade, government spending, and quality of medical treatment are significant macroeconomic factors affecting COVID-19 connectedness in different countries.

As is evident from the previous review, the network paradigm provides an insightful approach for studying and understanding the spreading of the pandemic within the context of the modern world’s connectivity. This approach goes beyond other non-network counterparts, which conceive interconnectedness either as an intrinsic property of countries^4,27,33^ or in terms of variables or indicators approximating aspects of cross-country interaction^35–37^. Therefore, using the network paradigm allows developing graph models representing interconnected structures at various geographical scales. However, there is still a long way to go on the network analysis of the interconnectedness of COVID-19 because current empirical approaches are relatively restricted compared to the broad range of human economic activity. In particular, the relevant research mainly applies to (a) social network models^45–51^, (b) supply and logistic networks^56,57^, and (c) networks of human mobility^53,54,60^. Moreover, due to big-data demand, relevant studies implemented at the global scale are considerably fewer. Toward responding to this demand, this paper focuses on the patterns and causes (first strand) of the COVID-19 spread. It develops a multidimensional methodological framework for understanding the spatio-temporal spread of the pandemic in the context of the global economy modeled as an interconnected cross-country structure. Therefore, this study goes beyond the previous complex network approaches by constructing a global network model incorporating dimensions of topology, geography, and economic openness. To do so, it conceptualizes worldwide interconnectedness based on economic globalization^61^ and, specifically, by constructing a network model of international tourism flows.

In terms of transport geography^62^ and spatial economics^63^, a tourism network belongs to the family of transportation networks. However, it is quite different from a typical transportation network, such as road, railway, maritime, or air transport network^43,64–66^, both in terms of structure and functionality. First, a tourism network has a specific economic configuration of its transport demand^64,67,68^, which involves movements for tourism purposes outside the country of residence. On the contrary, in a typical transportation network, the economic forces driving the transport demand are broad and unspecified since they may refer to trade, occupation, recreation, health, education, etc.^62,69^. Therefore, as far as the economic configuration of transport demand is concerned, a tourism network is well-defined within a unified functional framework (or economic activity), while a typical transport network (except a multilayer model) is not and is multivariable. However, in terms of modal configuration, a tourism network is multimodal because movements may occur through all transportation modes^62^. In contrast, a typical transport network usually is defined within a single transportation mode^43^. Finally, in terms of network topology^43^, a tourism network is more topological than geometric^64,67,68^, as a typical transportation network usually is. In particular, in a tourism network, edges are usually defined as accessibility links and not as routes or transportation channels^62,67^. Within this context, the network model of international tourism flows constructed in this paper suggests a good proxy^35,45,53^ for the outbreak of the pandemic because (a) it provides a comprehensive economic setting (tourism), (b) is integrated in terms of modal configuration, and (c) is more representative in terms of network accessibility.

This study builds on a three-dimensional conceptual model to analyze the worldwide spatio-temporal spread of COVID-19. It incorporates one dimension approximating the interconnectedness of the international tourist mobility, a second one describing the openness of countries to the globalized economy, and a third one expressing the spatial impedance to transportation. By constructing a single network model, this paper proposes an integrated framework for the study of the spatio-temporal spread of COVID-19. It also contributes to the literature with more realistic models of the worldwide interconnected system, where COVID-19 and other pandemics are spreading. The remainder of this paper is structured as follows: second section presents the methodological and conceptual framework of the study, third section shows the results of the analysis and discusses them within the context of regional and geographical sciences, and finally, in last section, conclusions are given.

## Methods

### Conceptual framework

**Figure 1:**
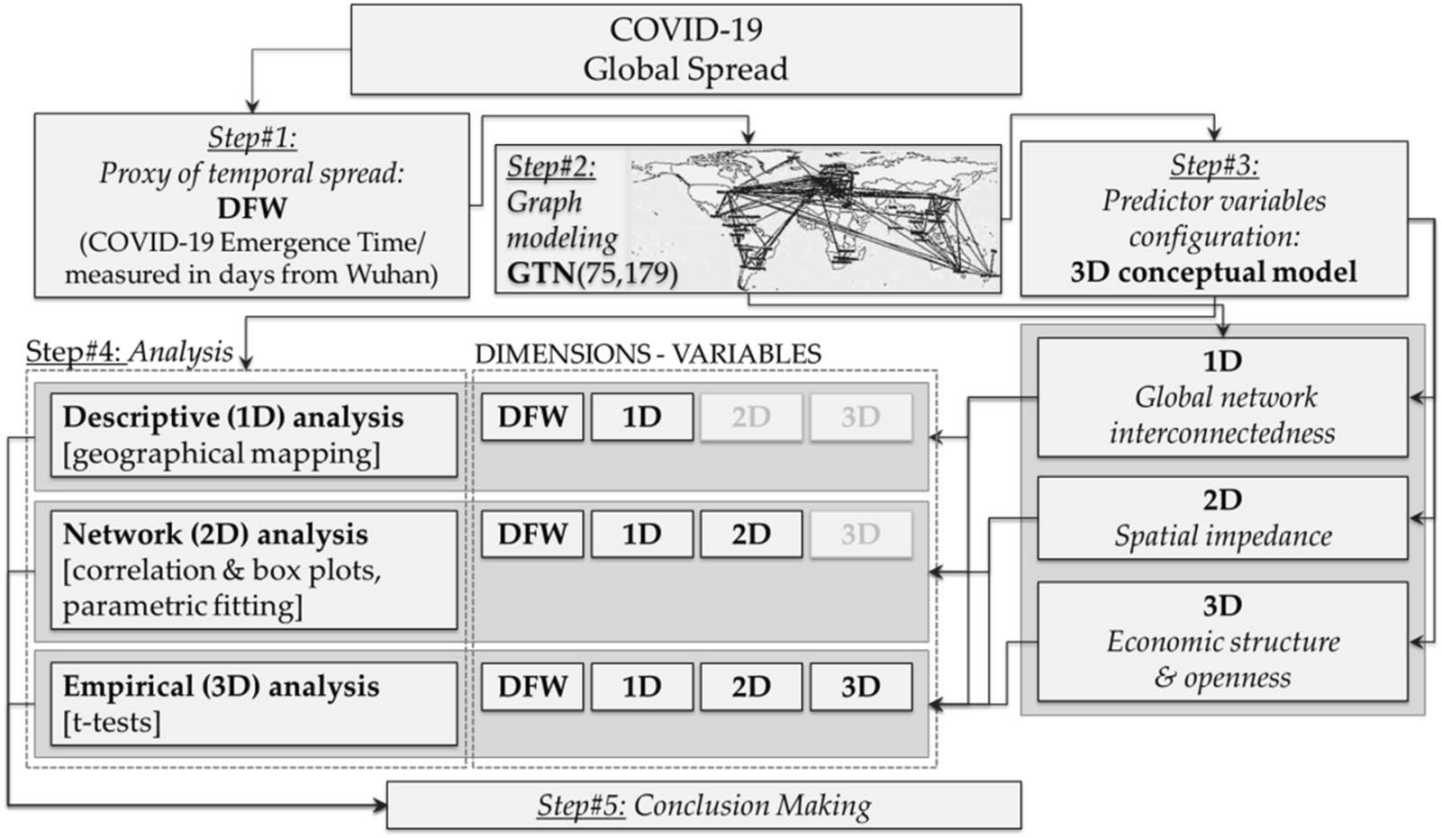
The conceptual framework of the study.

This paper develops a multidimensional (3D) model for understanding the uneven (cross-country) spread of COVID-19 in the context of the globally interconnected economy. The conceptual framework of the study is illustrated in Fig. 1 and consists of five steps. In the first step, we configure the variable expressing the temporal spread of the pandemic, measured in terms of the time at which the first infection emerged from Wuhan to each country (DFW). This variable provides a good proxy for interconnectedness because it quantifies the impedance of the pandemic flows between countries, namely the level at which each international link resists or facilitates the transmission of the pandemic flows in the network. In the second step, we construct a graph model of the Global Tourism Network (GTN), on which we compute fundamental (node) network measures. In the third step, the study develops a three-dimensional (3D) conceptual model to analyze the worldwide spatio-temporal spread of COVID-19. This 3D model includes different variables grouped by its dimensions. The first dimension (component) approximates the interconnectedness of international human mobility (1D: global network interconnectedness). The second one describes the spatial impedance to transportation (2D: spatial impedance).

The third dimension expresses the economic openness and attributes of countries in the structure of the globalized economy (3D: economic structure and openness). Variables belonging to the first dimension are configured from the available network measures of the GTN, whereas the other predictors extract from secondary databases. In the fourth step, the analysis applies toward different combinations (approaches) of the available dimensions. The first combination (DFW, 1D) expresses the embedding of variable DFW to the GTN’s space, as described by the spatial distribution of DFW in the GTN’s map. The second approach (DFW, 1D, 2D) is a network analysis incorporating structural and geographical information of the GTN. Finally, the third approach is an empirical analysis including all available information of the 3D model. In the final (fifth) step, we discuss the results and formulate conclusions. Overall, this 3D conceptual model examines if patterns in the cross-country temporal spread of COVID-19 relate to various aspects of interconnectedness in the structure of the worldwide mobility system. Within this context, it provides insights into the topological, geographical, and socioeconomic factors affecting the uneven spread of the pandemic.

### Graph modeling and analysis

**Figure 2:**
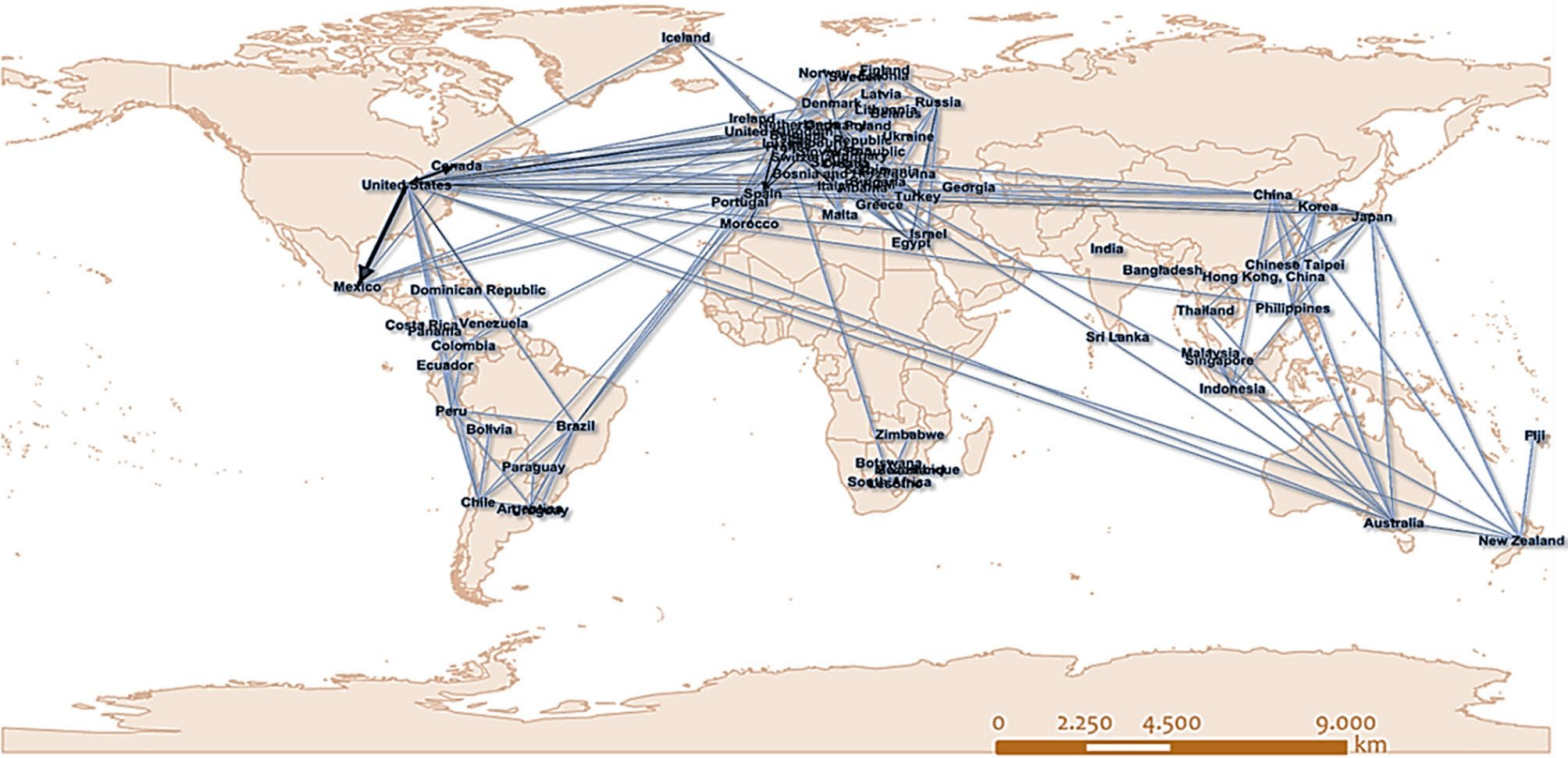
The graph model of the directed GTN (data of the year 2018, own elaboration based on ESRI ArcGIS 10.50; https://www.arcgis.com).

This study uses the network paradigm, as conceived by network science^42–44^, to represent the Global Tourism Network (GTN) into a graph. Generally, a graph model is a pair-set of nodes and edges^41^, quantitatively modeled using connectivity and weight matrices. In comparison with other models of socioeconomic or spatial interaction, graphs have the advantage of including, in a single model, both structural and functional information^70^, available both on a local (node, neighborhood) and global (the total network) scale. This property makes graph models more effective in describing real-world systems^71^ because it equips them with a double (hybrid) microeconomic and macroeconomic setting. The Global Tourism Network (GTN) is constructed on data of the year 2018, extracted from the Organization for Economic Co-operation and Development^72^. The available data include records of the top-5 markets (including either OECD or non-OECD countries) of the inbound and outbound tourism flows per OECD country. The GTN (Fig. 2) is a directed and weighted graph *G*(*V*,*E*), where nodes (*i*) correspond to tourism-destination countries, and links (*ij*) to the annual number of tourists originating from a node (country of origin) $i \in V$ and visited node $j \in V$ (destination country). The GTN is also a connected graph, composed of *n* = 75 nodes and *m* = 179 links (edges) and modeled in the *L*-space representation^43^, where nodes are connected if they are successive stops on a given route. Intuitively, the *L*-space (also called *space of stations*) resembles a physical representation because it illustrates direct connections between geo-referenced nodes but differs in the way that edges are shown, drawn as linear segments instead of real-shaped curves. This difference reduces modeling complexity, which is less costly than the physical representation because it requires just a pair of elements to display a connection (source node, target node). In general, the *L*-space representation is more geometric than others that are more topological (see^43,65^) and therefore is preferable for cases where the systems’ geometry matters. Within this context, the GTN is modeled in the *L*-space representation, where nodes are geo-referenced at the coordinates of the countries’ capital cities by using the Web Mercator projection^73^.

After constructing the graph model of the GTN, we compute fundamental network measures, as shown in Table 1. These measures are extracted from the relevant literature^41,43,74,75^ and capture different aspects of the GTN’s topology, such as connectivity, intermediacy, clustering, and accessibility.

**Table 1:** Measures of network topology used in the analysis of GTN.

Measure (symbol)	Description	Math formula	References
**Nomenclature**			
Graph	A pair set consisting of a node-set *V* and an edge-set *E* In graph *G*(*V,E*), *n* expresses the number of nodes and *m* the number of links	*G*(*V,E*)	^41,43^
**Network measures**			
Node degree (*k*)	The number of graph edges being adjacent to a given node *i* It expresses the communication potential of a node	$$\begin{gathered} {k_i} = m(i) = {m_i} = \sum\limits_{j \in V(G)} {{\delta_{ij}}} = \sum\limits_{j \in V(G)} {{\delta_{ij}}} , \, \hfill \\ {\text{where }}{\delta_{ij}} = \left\{ {\begin{array}{*{20}{c}} {1,{\text{ if }}{e_{ij}} \in E(G)} \\ {0,{\text{ otherwise}}} \end{array}} \right. \hfill \\ \end{gathered}$$	^41^
In-degree (*k–*)	The number of incoming edges being adjacent to given node *i* (applicable in directed graphs)	$$\begin{gathered} {k_i} - = {m_i} - = \sum\limits_{j \in V(G)} {{\delta_{ij}} - } ,{\text{ where}} \hfill \\ \, {\delta_{ij}} = \left\{ {\begin{array}{*{20}{c}} {1,{\text{ if }}{e_{ij}} \in E(G)} \\ {0,{\text{ otherwise}}} \end{array}} \right. \hfill \\ \end{gathered}$$	^41,43^
Out-degree (*k* +)	The number of outgoing edges being adjacent to given node *i* (applicable in directed graphs)	$$\begin{gathered} {k_i} - = {m_i} - = \sum\limits_{j \in V(G)} {{\delta_{ji}} - } ,{\text{ where}} \hfill \\ \, {\delta_{ij}} = \left\{ {\begin{array}{*{20}{c}} {1,{\text{ if }}{e_{ji}} \in E(G)} \\ {0,{\text{ otherwise}}} \end{array}} \right. \hfill \\ \end{gathered}$$	^41^
Node strength (*s*)	The sum of weights (*w*_*ij*_) of the links (*e*_*ij*_) being adjacent to a given node *i* The *δ*_*ij*_ operator is the Kronecker delta function yielding one for links belonging to graph *G*	$$\begin{gathered} {s_i} = s(i) = \sum\limits_{j \in V(G)} {{\delta_{ij}} \cdot {d_{ij}}} , \hfill \\ {\text{where }}{d_{ij}} = w({e_{ij}}){\text{ in km}} \hfill \\ \end{gathered}$$	^41,43^
Average degree $\left\langle k \right\rangle$	The mean value of node degrees *k*_*i*_, where *i* represents a network node	$\left\langle k \right\rangle = \frac{1}{n} \cdot \sum\limits_{i = 1}^n {k_i}$	^74^
Local clustering coefficient (*C*(*i*))	The probability a node *i* to have *E*(*i*) neighbors connected Computed on the number of triangles configured by node *i* to the number of the total triplets *k*_*i*_(*k*_*i*_–1) shaped by this node	$C(i) = \frac{E(i)}{{{k_i} \cdot \left( {{k_i} - 1} \right)}}$	^41^
Betweenness centrality (CB)	A proportion is defined by *σ*(*i*) shortest-paths that pass through node *i* to the total shortest-paths *σ* in the network It measures the intermediacy of network paths	$CB(i) = {{\sigma (i)} \mathord{\left/ {\vphantom {{\sigma (i)} \sigma }} \right. \kern-\nulldelimiterspace} \sigma }$	^75^
Closeness centrality (*CC*)	Computed on the average path-lengths *d*(*i*,*j*) originating from a given node *i* $\in$ *V* to all other nodes *j* $\in$ *V* in the network It is a measure of accessibility	$CC(i) = \frac{1}{n - 1} \cdot \sum\limits_{j = 1,i \ne j}^n {{d_{ij}}} = {\bar d_i}$	^75^
Eccentricity (*e*(*u*))	The longest path *p*(*u*,*j*) in the network from a given node *u* $\in$ *V*	$e(u) = \max \left\{ {\left. {p(u,j) \, } \right| \, j \in V} \right\} \,$	^75^

### The 3D model configuration

To study the international spread of COVID-19, we construct and collect twenty-four (24) variables, shown in Table 2. The first one includes epidemiologic information referring to the time-distance (measured in days from Wuhan—dfW) of the first confirmed case (infection) per country, whereas the other 23 variables group into the categories of the overall 3D conceptual approach. For the configuration of the variables included in the 1^st^ category (global network interconnectedness), the analysis employs graph modeling to represent the globally interconnected system of tourism mobility as a complex network. The variables included in the other two categories (2D: spatial impedance, and 3D: economic openness) originate from various Web sources of secondary data^72,73,76–83^, where cases only referring to the countries included in the GTN are included in the variables’ configuration. Within this context, all the available variables of Table 2 have length 75, with each element referring to a GTN node (country).

**Table 2:** Variables participating in the analysis of COVID-19 global spatio-temporal spread.

Group	Symbol	Description	Source/references
EPIDEMICS	*DFW*	COVID-19 emergence time: The time where the first COVID-19 infection emergence in a country. Is measured in days from Wuhan (dfW)	^82^
1D global network interconnectedness	*DEG*	Node degree: The number of connections per GTN node (country)	^41^*
	*IN.DEG*	Node in-degree: The number of incoming connections per GTN node	
	*OUT.DEG*	Node out-degree: The number of outgoing connections per GTN node	
	*STR*	Node strength: The sum of weights (tourists) of the (incoming and outgoing) connections per GTN node	
	*IN.STR*	Node in-strength: The sum of weights (tourists) of the incoming connections per GTN node	
	*OUT.STR*	Node out-strength: The sum of weights (tourists) of the outgoing connections per GTN node	
	*C*	Node clustering coefficient: The clustering coefficient per GTN node	^75^*
	*CB*	Node betweenness centrality: The betweenness centrality per GTN node	
	*CC*	Node closeness centrality: The closeness centrality per GTN node	
	*ECC*	Node eccentricity: The eccentricity per GTN node	
	*ECCFC*	Eccentricity from China: The eccentricity of a GTN node whether China is considered as the GTN’s center. Is defined by the relation *ECCFC*(*i*) = *ECC*(*i*)–3, yielding integer outcomes (where negative values imply that these cases are more central in reality than China in the GTN topology)	
2D spatial impedance	*CST*	Coastal indicator: Dummy (binary indicator) variable indicating whether a country is coastal (1) or not (0)	Own elaboration, based on^73^
	*DSTFC*	Distance from China: The shortest geographical distance of a country from China (measured in km)	
	*RDL*	Road length: The length of the road network in each country (measured in km)	^76^
	*RLL*	Rail length: The length of the rail network in each country (measured in km)	^80^
	*PRT*	Ports: The number of active ports in each country, for the year 2020	^83^
	*APRT*	Airports: The number of active airports in each country, for the year 2020	^79^
3D economic structure and openness	*GI*	Overall KOF Globalisation Index: Composite index measuring economic, social, and political globalization (yearly; from 1970 to 2017). Data refer to the year 2017	^77^
	*GDP*	Gross Domestic Product (GDP): GDP is the sum of gross value added by all resident producers in the economy plus any product taxes and minus any subsidies not included in the value of the products. It is calculated for the year 2017, without making deductions for depreciation of fabricated assets or depletion and degradation of natural resources. Data are in constant 2010 U.S. dollars	^81^
	*TFP*	Total factor productivity (TFP): Composite indicator expressing (loosely) the growth achieved due to labor and capital productivity factors. It is computed on constant national prices (2011 = 1)	^78^
	*POP*	Population: The number of citizens of the country according to the most recent national census	
	*HC*	Human Capital: Human capital index based on (a) years of schooling and (b) returns on education	
	*GDP.pc*	GDP per capita: The GDP divided by mid-year population. Data are in constant 2010 U.S. dollars	^81^
	*TFP.pc*	Total factor productivity per capita: The TFP, divided by the country’s population	^78^

*Own elaboration for the GTN, based on^72^ database for the year 2018.

### Quantitative tools and methods

This paper builds on a multidimensional network analysis employing methods of statistical mechanics, such as descriptive and statistical-inference analysis, parametric fitting, and non-parametric estimation methods^84–88^, to study the uneven spread of the COVID-19 pandemic. The descriptive methods used in the analysis are graphic methods aiming to display different aspects of distributions of the available data, either in a spatial context (spatial distribution maps, see^89^) or in a single-variable (boxplots plotting the median, *Q*_1_ and *Q*_3_ quartiles, and potential outliers and extreme values) or pair-wise (boxplots and scatter-plots plotting ordered pairs of numeric values corresponding to different variables) consideration^86,90^. In terms of statistical inference, the analysis builds on the formulation of error bars representing confidence intervals (CIs) constructed for estimating (at a 95% confidence level) the difference of the mean values between groups of cases within a variable^86^. These error bars graphically illustrate an independent samples *t*-test of the mean^90^. When they intersect with the zero-line (horizontal axis), the mean values of the groups cannot be considered statistically different, whereas when they do not intersect they can.

Parametric fitting techniques are applied to estimate the parametric curve that best describes the variability of the dataset displayed in a scatter plot. The available fitting curves examined in this part of the analysis are linear (1st-order polynomial, abbreviated Poly1), quadratic (2nd-order polynomial, Poly2), cubic (3rd-order polynomial, Poly3), one-term power-law (Power1), one-term Gaussian (Gauss1), one-term exponential (Exp1), and one-term logarithmic (Log1). All available types of fitting-curves can generally be described by the general multivariate linear regression model^86^:

1 $$\hat y = {b_1}{f_1}(x) + {b_2}{f_2}(x) + \ldots + {b_n}{f_n}(x) + c = \mathop \sum \nolimits^{b_i}{f_i}(x) + c,$$

where *f*(*x*) is either logarithmic *f*(*x*) = (log(*x*))^*m*^, or polynomial *f*(*x*) = *x*^*m*^, or exponential *f*(*x*) = (exp{*x*})^*m*^, with *m* = 1, 2, 3, or power *f*(*x*) = *a*^*x*^. The curve-fitting process estimates the *b*_*i*_ and *a* (where is applicable) parameters that best fit the observed data and simultaneously minimize the square differences ${y_i} - {\hat y_i}$^86^, as is shown in the relation:

2 $$\min \left\{ {e = \mathop \sum \limits_{i = 1}^n {{\left[ {{y_i} - {{\hat y}_i}} \right]}^2}} \right\} = \min \left\{ {\mathop \sum \limits_{i = 1}^n {{\left[ {{y_i} - \left( {\mathop \sum \nolimits^{b_i}{f_i}(x) + c} \right)} \right]}^2}} \right\}$$

The parameter estimation uses the Least-Squares Linear Regression (LSLR) method, based on the normality assumption for the differences $e\;\ N(0,{\upsigma }_e^2)$^86,90^.

Finally, the non-parametric kernel density estimation (KDE) method estimates the probability density function of a random variable. The KDE method returns an estimate $\hat f(x)$ of the probability density function for the sample data in a vector variable *x*. This estimate is based on a normal kernel function^84,85^ and is evaluated at equally-spaced (100 in number) *x*_*i*_ points covering the data’s range. In particular, for a uni-variate, independent, and identically distributed sample ***x*** = (*x*_1_, *x*_2_, …, *x*_*n*_), extracted from a distribution with unknown density (at any given point *x*), the kernel density estimator ${\hat f_h}(x)$ describes the shape of the probability-density function ƒ, according to the relation^84,85^:

3 $${\hat f_h}(x) = \frac{1}{n}\sum\limits_{i = 1}^n {{K_h}(x - {x_i})} = \frac{1}{nh}\sum\limits_{i = 1}^n {K\left( {\frac{{x - {x_i}}}{h}} \right)} ,$$

where *K* is the kernel (a non-negative) function and *h* > 0 is a smoothing parameter called bandwidth, which provides a scale (desirably the lowest possible *h*) in the kernel function *K*_*h*_(*x*) = 1/*h*·*K*(*x*/*h*) depending on the bias-variance trade-off dilemma^91^.

Overall, the multilevel analysis builds on statistical mechanics of the available network, socioeconomic, and geographical variables to conceptualize the worldwide uneven spatio-temporal spread of COVID-19 within the context of the global interconnected economy represented by the GTN.

## Results

### Descriptive (1D) analysis

**Figure 3:**
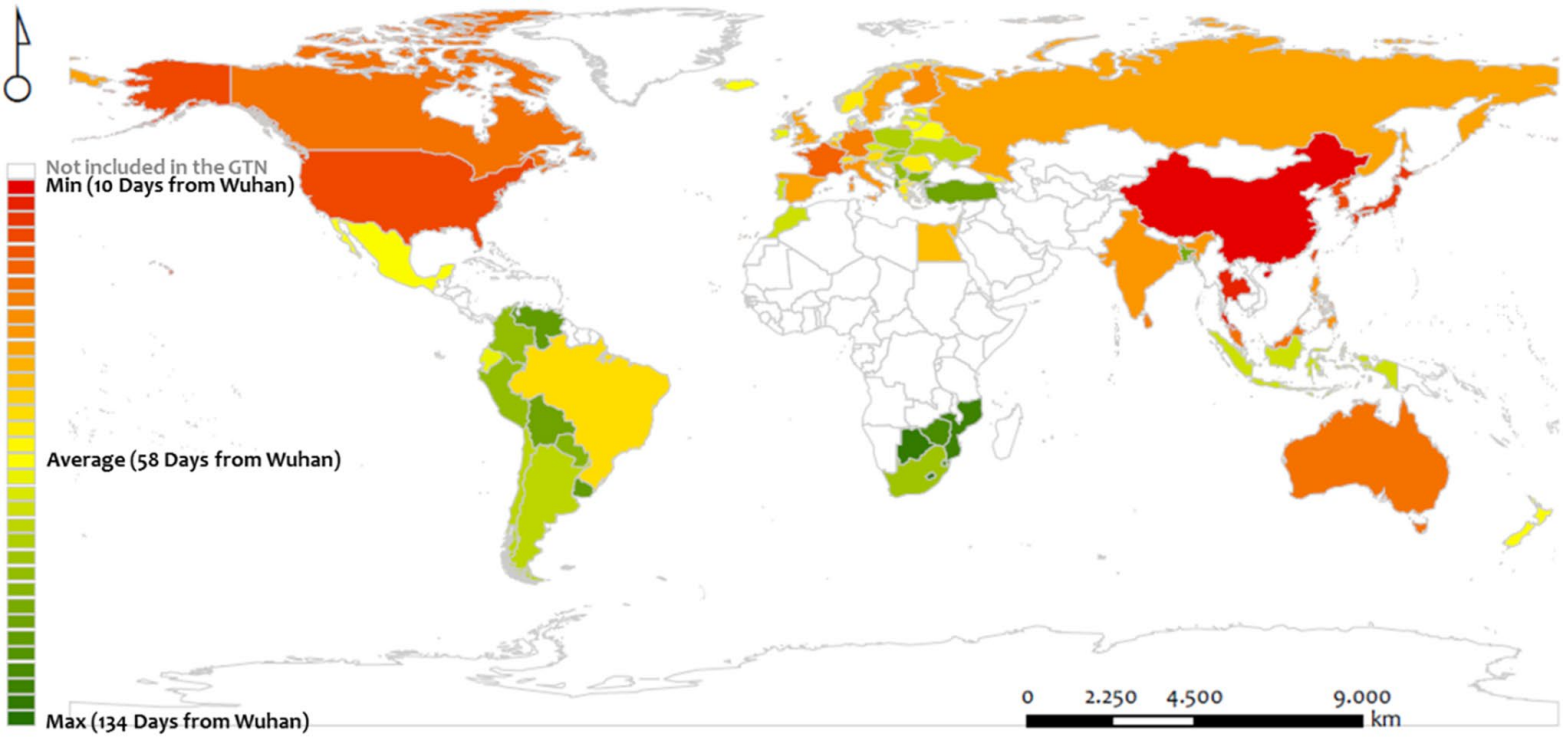
Heat map illustrating the spatial distribution of the temporal spread variable (*DFW*) expressing the number of days from Wuhan (dfW) since the first case emerged in a country (the days of the first infection per country), for the countries included in the GTN (own elaboration based on ESRI ArcGIS 10.50; https://www.arcgis.com).

At the first step of the analysis, we construct the heat map of Fig. 3, which shows the worldwide spatial distribution of the COVID-19 emergence per country (variable *DFW* expresses the number of days from Wuhan since the first infection). This heat map shows some clusters in the world map with distinguishable geographical patterns. The first cluster includes the red-colored countries, expressing cases where the first COVID-19 infection emerged relatively soon after the pandemic started in Wuhan (cluster of shortly infected countries). This cluster mainly includes countries neighboring China, along with North America, Australia, and Western Europe. The geographical distribution of this cluster configures a spatial pattern shaping an arc consisting of North America–Western Europe–Russian Federation–China–India–Thailand Islands–Australia, and covers the northern and eastern part of the world map.

The second cluster (Fig. 3) includes the green-colored countries, where the first COVID-19 infection emerged relatively far from the day the pandemic began in Wuhan. This cluster (of late infected countries) mainly distributes along the meridian zone, including (a) South America, Southern Africa, and Indonesia, (b) a sub-cluster of countries in Central Europe and the Western Mediterranean basin, and (c) Turkey. Finally, the third cluster includes the yellow-colored cases, which describe countries of average emergence of the pandemic from Wuhan (~ 58 days). This cluster configures a scattered spatial pattern including European and American countries, distributed along a southwest (in Latin America) and north (in Europe) line.

Overall, this descriptive analysis provides visual evidence about the global dynamics of the pandemic’s spread in the context of the GTN. As can be observed, proximity is evident in the distribution patterns of the COVID-19 spread. This observation complies with relevant findings^36,37,58^ about (a) the importance of geographical distance in the spread of the pandemic, and (b) the empirical knowledge stating that neighborhood connections undertake the highest traffic in spatial and transportation networks^43,62^. However, this is not the whole picture describing the spatial patterns in Fig. 3, which in such a case would follow just a circular distribution of color intensity. The clustered and asymmetric spatial distribution previously described in the heat map implies the effect of more forces than just proximity in the configuration of the COVID-19 spread, bringing into the light those theories about the socioeconomic factors determining transportation flows due to the differential demand (or attractiveness) emerging in space^62^. Therefore, this (1D) approach contributes to shaping an initial picture and motivates applying further research going deeper in the study of COVID-19 spatial spread.

### Network (2D) analysis

**Figure 4:**
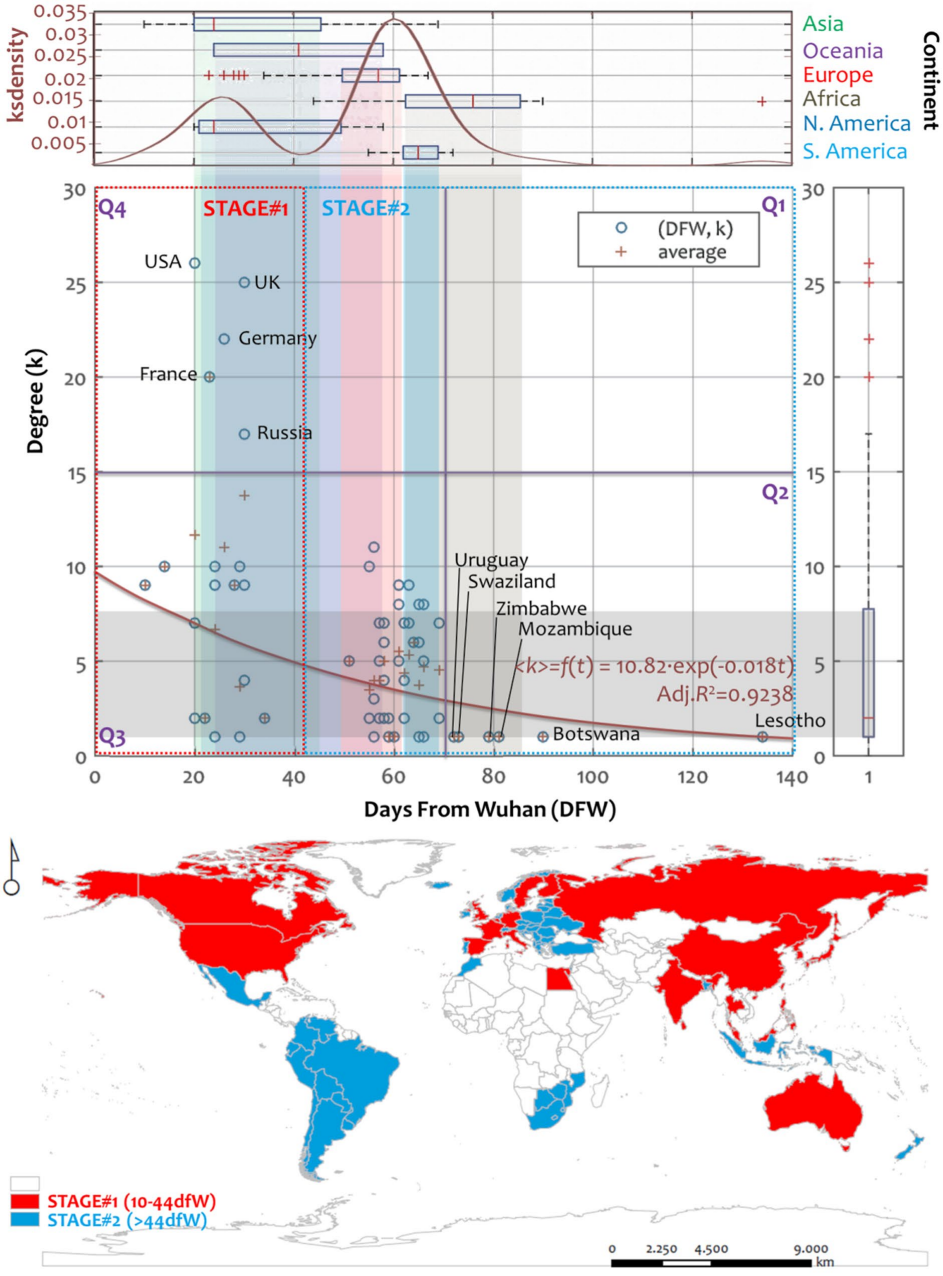
Multilayer scatterplot (*DFW*,*k*) showing the correlation between the days since the first infection from Wuhan (*DFW*) and the node-degree (*k*) of the GTN. Boxplots in each axis illustrate the distribution of each variable, where *DFW* further separates into continent groups. Shaded zones within the scatterplot express interquartile ranges of each boxplot. Quadrants *Q*_1_, *Q*_2_, *Q*_3_, and *Q*_4_ in the scatterplot area correspond to median lines. The fitting curve *f*(*x*) is applied to average degree values (< *k* >), expressed by cross “+” symbols. At the bottom, the map shows the spatial distribution of the two stages defined by the *ks*-density curve (maps are own elaboration based on ESRI ArcGIS 10.50; https://www.arcgis.com).

In the second part of the analysis, we construct a multilayer diagram including scatterplot, boxplots, and *ks*-density components (Fig. 4), to study the distribution of the pandemic’s emergence per country relative to the GTN network interconnectedness. The main window of the scatterplot (*DFW*, *DEG≡k*) shows the correlation between the days since the first infection from Wuhan (dfW) and the node-degree (*k*) of the GTN countries. At the axes, the boxplots illustrate the main aspects of the distributions (median, *Q*_1_ and *Q*_3_ quartiles, potential outliers, and extreme values) of the corresponding variables (*DFW* and *k*), where they further divide into continent groups in the horizontal axis (measuring days from Wuhan). In Fig. 4, according to the *ks*-density plot and the pattern of the scatterplot shown in the main window, we can observe two stages in the COVID-19 temporal spread throughout the GTN. These stages configure distinguished bell-shaped areas shown in the *ks*-density curve, defined by the cutting point of the 44th day from Wuhan (*t* = 44 dfW). The detection of these stages is due to the network configuration that applied a filter to the world countries keeping only those 75 belonging to the GTN. In particular, the first stage includes nodes infected before the 44th day from Wuhan (≤ 44 dfW), mainly described by the outbreak in Asia and North America (as is evident by the country boxplots). The second one includes nodes infected after the 44th day from Wuhan (> 44 dfW), described by the outbreak in Europe, South America, and Africa. The outbreak in Oceania spreads along both stages but is slightly positively asymmetric, having its median value placed at the first stage. This outcome complements and revises with broader information the finding of the authors of^13^, who observed three clusters in the COVID-19 global spread, following a route from China to West Asia, Europe, North America, and South America. Although the pandemic emerged in Europe mainly in the second stage, the cases of the UK (*k* = 25), Germany (*k* = 22), France (*k* = 20), and the Russian Federation (*k* = 17) faced COVID-19 in the first stage. All these European countries are GTN hubs (nodes of high degree) and belong to the *Q*_4_ quartile (*t* ≤ 44 dfW, *k*_*i*_ > 15), as is shown in Fig. 4.

On the other hand, the late infected nodes mainly concern African countries belonging to the *Q*_2_ quartile (*t* > 44 dfW, *k*_*i*_ ≤ 15) and, in terms of the GTN connectivity, they are spokes, namely nodes of one connection, with degree *k* = 1. According to the *ks*-density distribution, most nodes (> 85%) faced the pandemic between the 20th and the 70^th^ dfW. The interquartile range (50% of data) at the period is 30–64th dfW. A parametric fitting curve applies to the average degree (< *k* >) data to shape a picture of how average connectivity behaves as a function of the COVID-19 emergence time (*t* = *DFW*) in the GTN. The (adjusted) coefficient of determination (*R*^2^ = 0.924) shows a high correlation < *k* >  = *f*(*t*) between these two variables, described by a decaying exponential pattern with mathematical expression < *k* >  = *f*(*t*) = 10.82·exp(− 0.018*t*). This exponential decay pattern implies that, on average, the GTN hubs are early infected by the pandemic (which is also verified by the fact they belong to the first stage), while lower degree nodes were late infected. In general, this fitting curve, along with the multilayer scatterplot, shows that the relationship between interconnectedness in the GTN and the COVID-19 emergence is not likely to be a result of randomness, implying that network interconnectedness is related to the temporal spread of the pandemic within a causative context. This finding provides a context quantitatively defining the relationship between global interconnectedness and COVID-19 spread. This context can support relevant studies observing that international connectivity is determinative to the pandemic's outbreak^45,48^.

**Figure 5:**
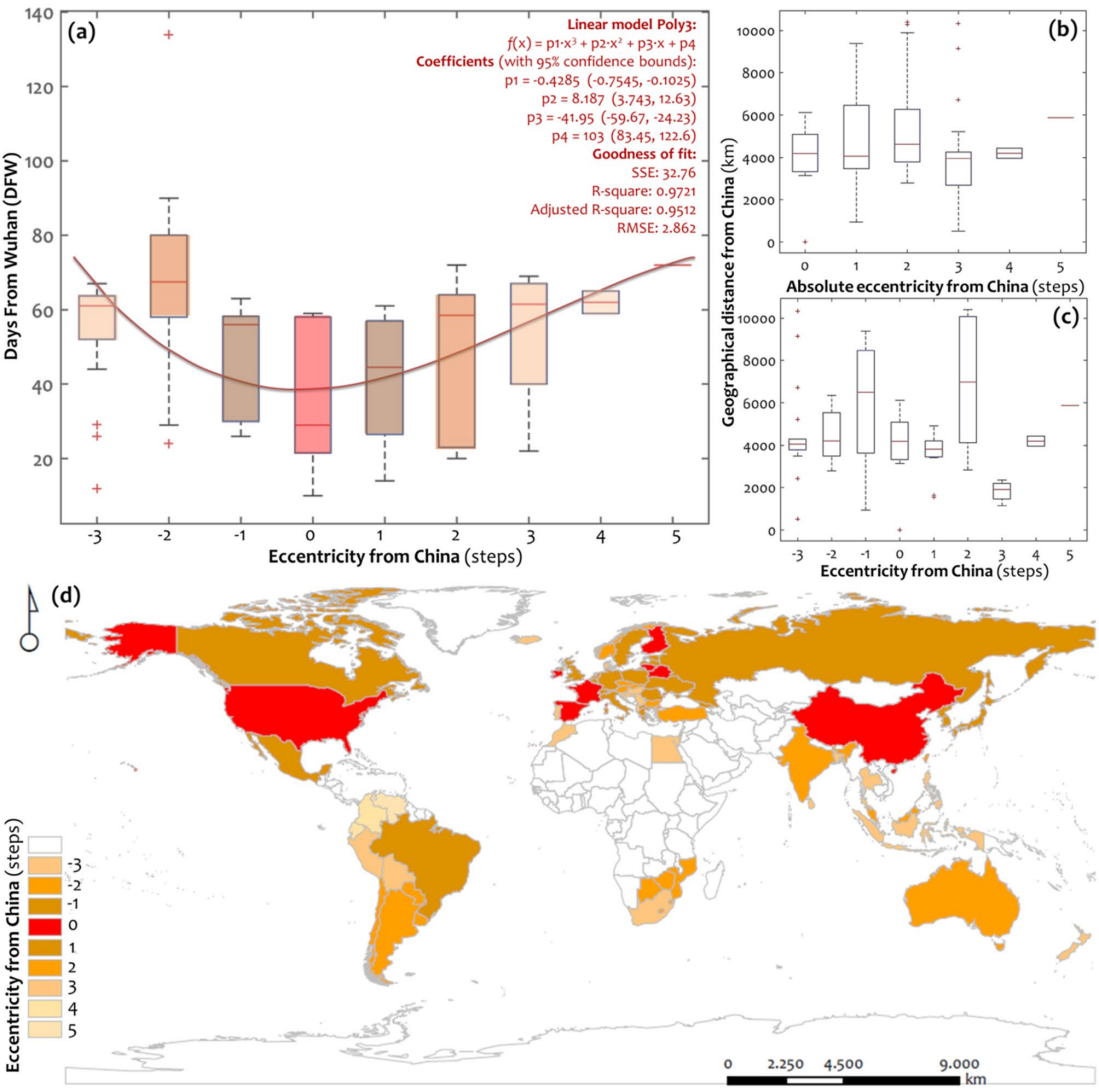
Boxplots expressing (**a**) the days since the first infection from Wuhan (DFW) for each class of the GTN’s eccentricity centered at China (abbreviated: eccentricity from China), the eccentricity of which is 3 steps (also shows the fitting curve of best determination applied to average values), and the geographical distance from Wuhan (DFW) for each class of (**b**) absolute and (**c**) non-absolute eccentricity from China. Also, the diagram shows the fitting curve of best determination applied to the average values. The bottom map (**d**) shows the spatial distribution of the eccentricity from China (maps are own elaboration based on ESRI ArcGIS 10.50; https://www.arcgis.com).

In geographical terms, the map in Fig. 4 illustrates the spatial distribution of the two stages of COVID-19’s temporal spread in the GTN. As can be observed, the first stage of the pandemic’s temporal spread mainly covers the northern hemisphere, whereas the second stage covers the southern hemisphere, with notable exceptions the cases of Central Europe and Australia, respectively. As is evident from the previous analysis, the spatial patterns of the two-stage worldwide temporal spread of COVID-19 in the GTN appear as more a matter of network interconnectivity (node degree) than of spatial proximity. This observation complements these works focusing either on the importance of geographical distance^36,55,58^ or on the importance of international connectivity^45,48,53^ in the spread of the pandemic, and develops a common context for the study of the pandemic’s outbreak. The boxplots of Fig. 5 are constructed to study in more detail the effect of proximity in the temporal spread of the pandemic throughout the GTN structure. The boxplots illustrate how the variables of COVID-19 emergence time (Fig. 5a), measured in days since the first infection from Wuhan, and spatial (geographical) distance (Fig. 5b,c) distribute along groups configured by the node-eccentricity of the GTN. To provide a reference to the case of China, due to its importance in the spread of the pandemic, we center the node-eccentricity to China by subtracting all scores by China’s eccentricity, which equals 3 steps. Therefore, we compute a new variable named “eccentricity from China” (*ECCFC*), defined by the algebraic difference *ECCFC*(*i*) = *ECC*(*i*)–3 according to Table 2, where *i* is a GTN node. Although *ECCFC* measures distance, its algebraic definition also allows receiving negative integer values, implying that these cases are more central in reality than China in the GTN topology. For the sake of completeness, we also consider in the analysis the absolute of the *ECCFC* variable.

In descriptive terms, the boxplots of Fig. 5 illustrate the correlations of the pairs of variables (*DFW*, *ECCFC*), (*DSTFC*, *ECCFC*), and (abs(*DSTFC*), *ECCFC*). As is evident in Fig. 5a, the curve fitting applied to the boxplot medians of the eccentricity groups shapes a cubic pattern, which describes the median-data variability under a high level of determination (adj.*R*^2^ = 0.9512). This “U”-shaped pattern yields a global minimum at the value of *ECCFC*(*i*) = 0, which implies that, on average, the countries where the pandemic first emerged are those with the same node-eccentricity as China, in the GTN. In all the other cases (nodes), the values of COVID-19 emergence time shape and almost symmetric distribution along both sides of the group of China’s eccentricity. Although this pattern regards averages (and more accurately, to the extent that the median-values are representative of the cases included in a boxplot), this observation implies that the center of the spread of the pandemic in the GTN was not only China, but the core of countries having the same score of eccentricity as China. In other words, it implies that the center of the pandemic spread includes all these nodes (countries) that are as central in this network as China is.

As can be observed in the map of Fig. 5d, these countries, belonging to the eccentricity-core of China, are not described by geographical proximity with China, but they are as central as China in the GTN. This finding is striking because it proposes reconsidering the certain central role of China^2,23,27,36,61^ in the spread of the pandemic within the context of network connectivity. In particular, this result suggests that China has played a critical role in the virus spread not because of the country's first outbreak of COVID-19 worldwide, but because of the country's importance to the network connectivity, as a hub. This observation is further supported by the results of Fig. 5b,c , showing that spatial proximity does not seem to be particularly related to the temporal spread of the pandemic (*DFW*) because curve-fitting does not yield any pattern with considerable determination (*R*^2^ > 0.5). Intuitively, the median-values arrangement in the boxplots in Fig. 5b,c approximates an almost linear pattern in parallel to the horizontal axis, which might imply that the temporal spread of COVID-19 is indifferent to geographical proximity. In this part, the overall approach highlights the importance of network centrality and thus the critical role of hubs (as China is) in the worldwide temporal spread of the pandemic (to the extent that centrality is described by the metric of eccentricity, measuring central positioning in the network).

### Empirical (3D) analysis

**Figure 6:**
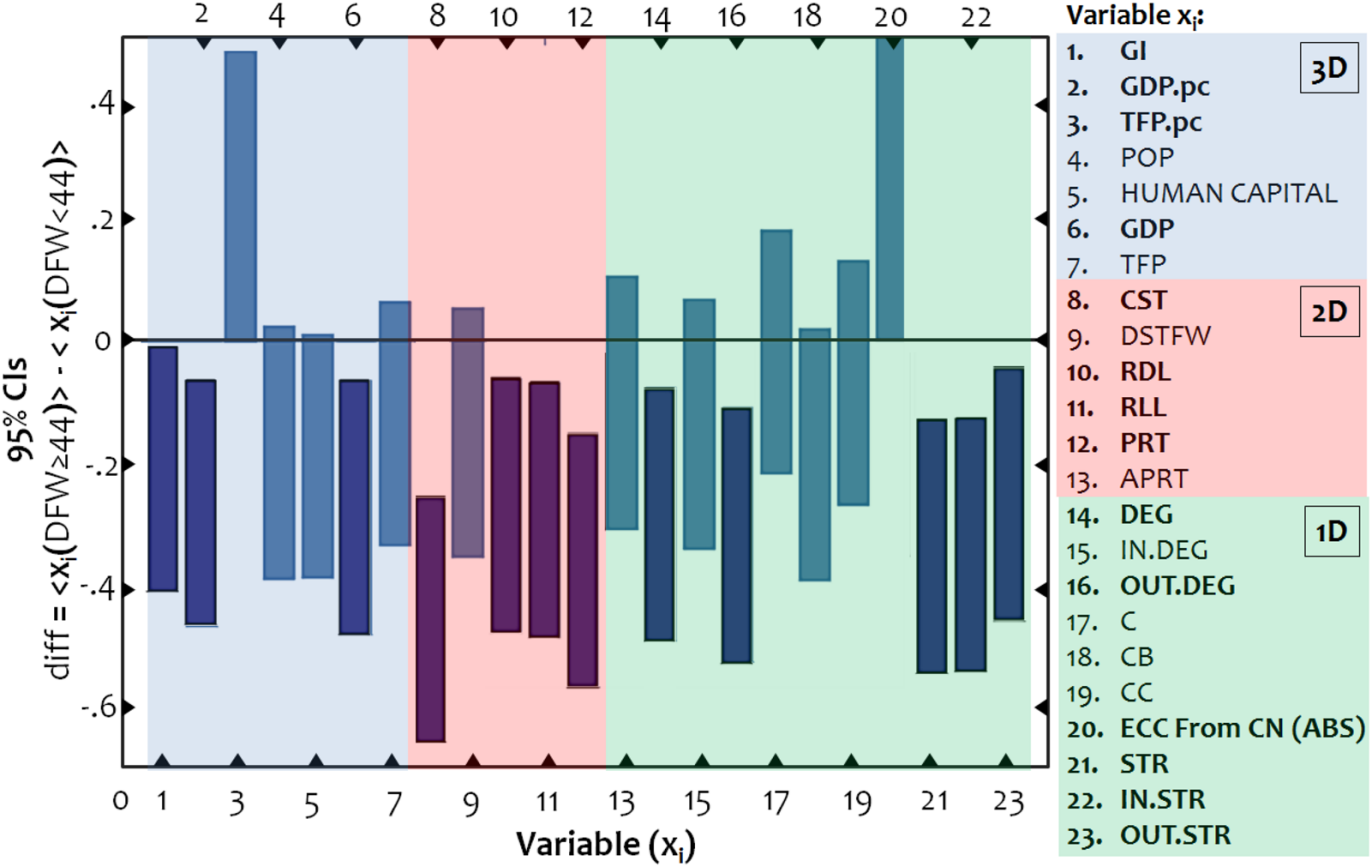
Error bars of 95% confidence intervals (CIs) for the average differences $\left\langle {{\text{diff(}}{x_{\text{i}}}{)}} \right\rangle$, computed on standardized variables (ranging from 0 to 1) between the groups of cases ***x***(*t* < 44DFW) and ***x***(*t* ≥ 44DFW) defined by the cutting value *t* = 44DFW (the number of days since the first infection in Wuhan), for each of the available network and economic variables (***x***_1_, ***x***_2_, …, ***x***_19_). Labels shown in bold font have significant differences.

In the final step of the analysis, we examine which variables included in the 3D-conceptual model of Table 2 can be considered significant determinants for the worldwide spatio-temporal spread of COVID-19, in the GTN. To do so, we apply a series of *t*-tests to compare the means between the groups defined by the two stages of the temporal spread of the pandemic. For better supervision of the results, the error bars shown in Fig. 6 visualize the t-tests, where each variable is standardized to the interval [0,1] so that the results of the *t*-tests are comparable. When the error-bars intersect with the horizontal axis (zero-line), the group mean values can be considered statistically equal, under a 95% certainty. When error bars do not intersect the zero-line, they can be considered statistically different (one group performs better than the other).

As can be observed in Fig. 6, in terms of network interconnectedness (1D conceptual component), the GTN nodes (countries) belonging to the first stage of temporal spread are cases with a higher (a) degree (variable *DEG*), expressing the number of connections of a node in the GTN; (b) outgoing degree (variable *OUT.DEG*), expressing the number of outgoing connections of a node in the GTN; (c) absolute eccentricity from China (variable *EECFC*(*ABS*)), expressing the network binary distance from China; (d) strength (variable *STR*), expressing the sum of incoming and outgoing tourists a GTN-node annually mobilizes, (e) incoming strength (variable *IN.STR*), expressing the sum of incoming tourists a GTN-node annually receives; and (f) outgoing strength (variable *OUT.STR*), expressing the sum of outgoing tourists a GTN-node annually sends to other destinations. The *t*-tests applied to the variables of this conceptual component indicate that network interconnectivity and central structure are significant determinants in the early global temporal spread of COVID-19.

In terms of spatial impedance (2D conceptual component), the GTN countries belonging to the first stage of temporal spread are cases (a) with more coastal geomorphology (variable *CST*), (b) they have larger road (variable *RDL*) and rail lengths (variable *RLL*), and (c) a greater number of ports (variable *PRT*) than those included in the second stage of temporal spread. An interesting “insignificant” result in this conceptual group is the variable APRT describing the number of airports included in the GTN countries. At a glance, this observation opposes the literature findings^45,53,54,58^ stating that air travel and connectivity are major determinants of the pandemic's spread. However, in conjunction with the significant *t*-tests observed for the degree (*DEG*) and strength (*STR*) variables (included in the network interconnectedness group), the insignificant performance of the APRT variable implies that the airport network becomes a significant determinant in terms of connectivity rather than of infrastructure capacity. Regardless of the participation of the APRT variable, the *t*-tests applied to the variables of the 3d conceptual component illustrate how land and maritime transport capacity (which are critical aspects of transport integration) significantly affected the early temporal spread of the pandemic worldwide.

Finally, in terms of economic openness (3D conceptual component), the GTN countries belonging to the first stage of temporal spread have a higher (a) globalization index (variable *GI*), (b) GDP and GDP per capita (variable *GDP.pc*), and (c) total factor productivity per capita (variable *TFP*.*pc*) than those included in the second stage of the COVID-19 temporal spread in the GTN. These results come in line with those works observing the importance of globalization on the spread of the pandemic^2,58,61^ and with others identifying productivity as a major pandemic spreader^2,48^. Generally, the *t*-tests applied to the variables of this conceptual component show that the countries with higher economic openness (those more integrated into the globalized economic structure) were subjected earlier to the pandemic than those of lower economic openness. Overall, the *t*-test analysis provides an integrated framework for understanding the uneven spread COVID-19, showing that network interconnectedness, economic openness, and transport integration are key determinants in the early global temporal spread of the pandemic.

## Conclusions

This paper developed a multilevel methodological framework for understanding the uneven spatio-temporal spread of COVID-19 in the context of the globally interconnected economy. The framework is built on a three-dimensional conceptual model, incorporating one dimension for approximating the interconnectedness in worldwide human mobility, a second one for the global economic openness, and a third one for the spatial impedance to transportation. The analysis applied to a major temporal variable expressing the pandemic’s emergence (measured in days from Wuhan since the first infection) and to another twenty-three variables grouped into three categories (dimensions) of a 3D conceptual model. Firstly, the descriptive (1D) analysis revealed three clusters in the world map with distinguishable geographical patterns of the pandemic temporal spread. The first one of early infected countries configured a geographical arc distributed throughout the countries neighboring China, North America, Australia, and Western Europe. The second cluster of late-infected cases was distributed mainly along the meridian zone of South America, Southern Africa, and Indonesia, including a sub-cluster with countries of Central Europe, the Western Mediterranean basin, and Turkey. The third cluster configured a scattered spatial pattern throughout the globe. The network (2D) analysis led to further specialization of these findings and revealed two main stages in COVID-19’s temporal spread throughout the GTN. The first included nodes infected by the pandemic before the 44th day from Wuhan (≤ 44 dfW) and described the outbreak in Asia and North America. The second one included nodes infected after the 44th day from Wuhan (> 44 dfW) and described the outbreak in Europe, South America, and Africa. Finally, the outbreak in Oceania spread along both stages. In geographical terms, the first stage of the pandemic’s temporal spread appeared to be more a matter of the northern hemisphere, whereas the second stage involved the southern hemisphere. Next, the analysis applied to the average degree and temporal spread of the pandemic showed a decaying exponential pattern, indicating that hubs in the GTN were early infected, while lower degree nodes were infected late by the pandemic. This pattern showed that network interconnectedness is related to the temporal spread of COVID-19 within a causative context. Further, the network analysis revealed a “U”-shaped pattern describing the correlation between network eccentricity and the temporal spread of the pandemic, describing that the center of the pandemic’s temporal spread in the GTN included China and the core of countries that are as central in the network as China is. This finding is striking, implying that the importance of China in the spread of the pandemic is more a matter of its hub connectivity in the GTN than the worldwide first emergence of the pandemic in the country. The analysis also revealed that spatial proximity was not a major determinant of the temporal spread of the pandemic. Finally, the empirical (3D) analysis applied to the total set of available variables indicated first that network interconnectivity and central structure are significant determinants of the early temporal spread of COVID-19, secondly that countries with higher economic openness earlier submitted to the infection of the pandemic, and finally that land and maritime transport integration significantly affected the early temporal spread of the pandemic worldwide. Overall, this paper (a) revealed two major stages in the temporal spread of the pandemic within the context of interconnected worldwide mobility networks, (b) highlighted the importance of network centrality in the worldwide temporal spread of the pandemic, (c) showed that network interconnectedness, economic openness, and transport integration are key determinants in the early global spread of the pandemic, and (d) revealed that the spatio-temporal patterns of the worldwide spread of COVID-19 were more a matter of network interconnectivity than of spatial proximity. These findings can provide useful insights not only for contributing to the scientific knowledge but also for promoting current and future practices in epidemiology and public health management. For instance, the mix and intensity of policy measures (personal protection; self and social isolation; local and national lockdowns; etc.), aiming to support strategies against current or future waves of the (or a) pandemic, can differentiate per country according to the tradeoff between the specific topological (connectivity), economic (openness), and geographical (distance) attributes of the country in the spreading network. Provided that time is extremely costly during the outbreak of a pandemic, countries with a more central topological position in a virus spreading network should be more alerted and apply faster more severe measures than others, regardless of the geographical distance of the country from the pandemic’s source. To do so, the success in the mix of such measures depends on the profound knowledge about the position of a country in its network and economic environment, toward which this paper contributes to a better understanding.
